# 

**Published:** 1849-04

**Authors:** 


*Buffalo Medical College.*—The annual commencement at this Institution is on the 18th inst. The valedictory address will be delivered by Prof. C. A. Lee. The commencement exercises will take place in the evening, and not in the forenoon, as hitherto.

---

☞ Dr. Green's communication will appear in our next.

